# Children with oedema recover better than those with severe wasting in outpatient therapeutic program at Boloso Sore district, Southwest Ethiopia

**DOI:** 10.1186/s13104-018-3232-x

**Published:** 2018-02-09

**Authors:** Mulugeta Yohannis Kabalo, Bereket Yohannes

**Affiliations:** 1 School of Public Health, Wolaita Sodo University, P.o.box 126, Wolaita Sodo, Ethiopia; 2 Damot Pulasa District, Wolaita Sodo, Ethiopia; 3 School of Public and Environmental Health, Hawasa University, Hawasa, Ethiopia; 4 Centre for International Health, The University of Bergen, Bergen, Norway

**Keywords:** Oedema, Severe acute malnutrition, Treatment outcome

## Abstract

**Objectives:**

Severely undernourished young children clinically present with a typical nutritional oedema or none-oedematous. However, research evidence is limited on how these types predict treatment outcomes in Ethiopia. This study was aimed to compare oedematous and none-oedematous children for their treatment outcomes in Boloso Sore district in Southwest Ethiopia.

**Results:**

The overall recovery rate was 396 (68%). From oedematous children; 235 (79.9%) recovered, 18 (6.1%) transferred, 6 (2.0%) defaulted, 3 (1.0%) died, and 32 (11%) remained none-respondents. The treatment outcomes among the none-oedematous children were 161 (55.9%), 12 (4.2%), 4 (1.4%), 3 (1.0%), and 108 (37.5%) in similar order. Treatment outcomes of severely undernourished children in the two arms were statistically different (Χ^2^ = 5.82, P < 0.016). Severely malnourished children with oedema were 2.3 times highly likely to recover as compared to those without it (adjusted hazard ratio = 2.3 at 95% confidence interval: 1.79, 2.82). We documented that oedematous children in the study area had a better likelihood of recovery as compared to those with severe wasting. We recommend targeted community outreach activities on severe acute malnutrition focusing on the types.

**Electronic supplementary material:**

The online version of this article (10.1186/s13104-018-3232-x) contains supplementary material, which is available to authorized users.

## Introduction

Severely malnourished children are about 10 times at higher risk of death than those not affected [1, 2]. Over 13 million children aged less than 5 years are affected with severe acute malnutrition (SAM) in low income countries; its case fatality rate in this region is of great burden [1, 2]. The ‘Outpatient Therapeutic Program (OTP)’ has been implemented in many countries in response to the burden of SAM. The program was intended to decentralize treatment of severe acute malnutrition (SAM) [3–5].

Based on the existing protocol, severely wasted children with weight for height ‘z-score’ less than − 3 or mid upper arm circumference (MUAC) less than 115 mm, and those children with bilateral pitting oedema to legs are considered as severely malnourished. Severely malnourished children with medical complications are treated as inpatient and those without medical complications are treated in OTP [6–8]. The complications include diarrhoea, coughing, dehydration, vomiting, anorexia and related medical symptoms.

The SAM affected children in OTP receive ready to use therapeutic foods (RUTF) and supportive medications for 2 months. In Ethiopia, this service is given through community health posts [3–5]. A recovery from SAM is declared in either of these conditions: a 15% weight gain for children with severe wasting and if oedema is lost for 2 consecutive weeks for those with oedema on admission [3–5]. Oedematous SAM has been a concern in low income settings resulting in high case fatality. None-oedematous SAM also causes mortality in children. Both forms are common among children less than 5 years of age in low income settings. Reviews indicate differences in pathogenesis, and aetiology of oedematous and none-oedematous SAM. Besides, there is evidence that caregivers of oedematous children are more concerned for their child nutritional disorder than caregivers of none-oedematous children [5, 9–12].

The existing SAM management protocol allows both forms of SAM (oedematous and none-oedematous) to be treated in OTP through RUTF [13, 14]. Some studies indicate the presence of association between treatment outcomes of SAM and the type of malnutrition [1, 15–17]. However, the available evidences on treatment outcomes of SAM by type of malnutrition are very limited. Therefore, we compared the treatment outcomes of SAM among oedematous and none-oedematous children treated in OTP at Boloso Sore district in South Ethiopia.

## Main text

### Method

This study was done at Boloso Sore district in Southwest Ethiopia. It is located in 300 km distance in south direction from Addis Ababa. It is a densely populated area experiencing repeated episodes of severe food scarcity [18]. The district has a town administration and 30 rural kebeles. The people get health service from a hospital, 6 health centres and 32 health posts in the area [19]. Thirty-two OTP sites give treatment to severe malnutrition children at outpatient in the study area. According to the local health department reports, 12,000 children under age 5 were treated at community outpatients at therapeutic centres in the area during 2014 and 2015.

We did a retrospective cohort study on children OTP records in the 2 years (2014–2015). All children admitted to OTP in Boloso sore district with SAM during the 2 years were the source population. Records were excluded when the child’s age and sex, and admission criteria were not registered in OTP cards.

The sample size was calculated assuming an anticipated proportion of the outcome (a recovery rate of 37.1%), a 95% confidence level, an 80% power, a relative risk by the type of undernutrition RR = 0.415, a design effect of 2, and a 10% consideration for missing values [8, 20]. The computed sample size was 642 children records in OTP. We purposely selected 6 sites from 32 OTP centres based on higher case load. The required sample was then allocated for each site proportionally. Children OTP cards were randomly selected from each sites. The data were retrieved by trained enumerators using pretested tool. The study tool was developed from OTP card formats and was pre-tested in other OTP sites before use.

Data were entered, cleaned, coded and analyzed in SPSS version 20 [21]. The treatment outcomes were calculated and compared with sphere standard yardsticks. The disparity in the treatment outcomes among oedematous and none-oedematous children were compared using Chi square (Χ^2^), and P < 0.05 was used to state statistical significance. The effect size was estimated with adjusted hazard ratio (AHR) at 95% CI and the survival status was reported using Kaplan–Meier survival curve.

### Operational terms

#### Oedematous and none-oedematous children

Children who were admitted to OTP with the diagnoses of nutritional oedema were considered as oedematous and those who were admitted to the program with severe wasting were categorized as none-oedematous.

#### Recovered

If severely wasted children gained 15% of their weight after admission, and if those oedematous lost the swelling after 2 consecutive weeks of admission they were classified as recovered.

#### Not recovered

Children who were discharged from OTP with any outcome except recovery were termed as not recovered. A none-recovered child could be died, defaulted, none-responder or medical transfer.

#### Defaulter

A SAM case absent for 2 consecutive weeks after getting admitted to OTP and confirmed as alive by home visit were reported as defaulter.

#### None-respondent or none-responder

A SAM case that did not reach the discharge criteria after 2 months stay in OTP was classified as a none respondent or none-responder in this study.

### Result and discussion

#### Socio-demographic characteristics and nutritional status at admission

A total of 582 (90.6%) OTP cards of children were analysed. Sixty (9.4%) cards were excluded due to missing main records. Of those included in the analysis, 288 (49.5%) were none-oedematous and 294 (50.5%) had oedema. Children aged ≤ 24 months were 207 (72%) in none-oedematous study arm and were 212 (72.1%) in the oedematous study arm (Additional file 1).

#### Recovery rate and other treatment outcomes at OTP

The overall recovery rate of children admitted to OTP was 396 (68%). From oedematous arm 235 (79.9%) with 95% CI 75.5, 84.4 children recovered, 3 (1.0%) died, 6 (2.0%) defaulted, 18 (6.1%) were transferred elsewhere for medical reason, and 32 (10.9%) were none-respondents. Conversely, of those with oedema 161 (55.9%) with 95% CI 50.3, 61.8 recovered, 3 (1.0%) died, 4 (1.4%) defaulted, 12 (4.2%) medically transferred and 108 (37.5%) were none-respondents (Table 1). None-oedematous children gained mean (SD) weight of 3.9 (2.23) g/kg/days. Both weight gain and recovery rate of none-oedematous children in the study area were intolerable based on international sphere standard.

**Table 1:** Treatment outcomes of oedematous and none-oedematous children treated at OTP

Performance indicators	Outcome status and other indicators	
	Oedematous children (%)	Non-edematous children (%)
Recovery rate	235 (79.9)	161 (55.9)
Death rate	3 (1.0)	3 (1.0)
Default rate	6 (2.0)	4 (1.4)
Medical transfer	18 (6.1)	12 (4.2)
None respondents	32 (10.9)	108 (37.5)
Average length of stay in weeks (95% CI)	6.4 (6.10, 6.70)	8.1 (7.77, 8.51)

#### Variation in treatment outcomes by type of the SAM

Statistically significant variation in treatment outcomes was observed between the study arms; the types of SAM at admission (none-oedematous and oedematous) (Χ^2^ = 5.82, P = 0.016). Similar findings were reported from the south [22] as well as Northern part of Ethiopia [20]. A study in Southwest Ethiopia at Jima reported no association between the types and treatment outcomes of SAM in stabilization centre [17]. The variations might be explained by the difference in the study settings and the type of facilities the care was given.

**Fig. 1:**
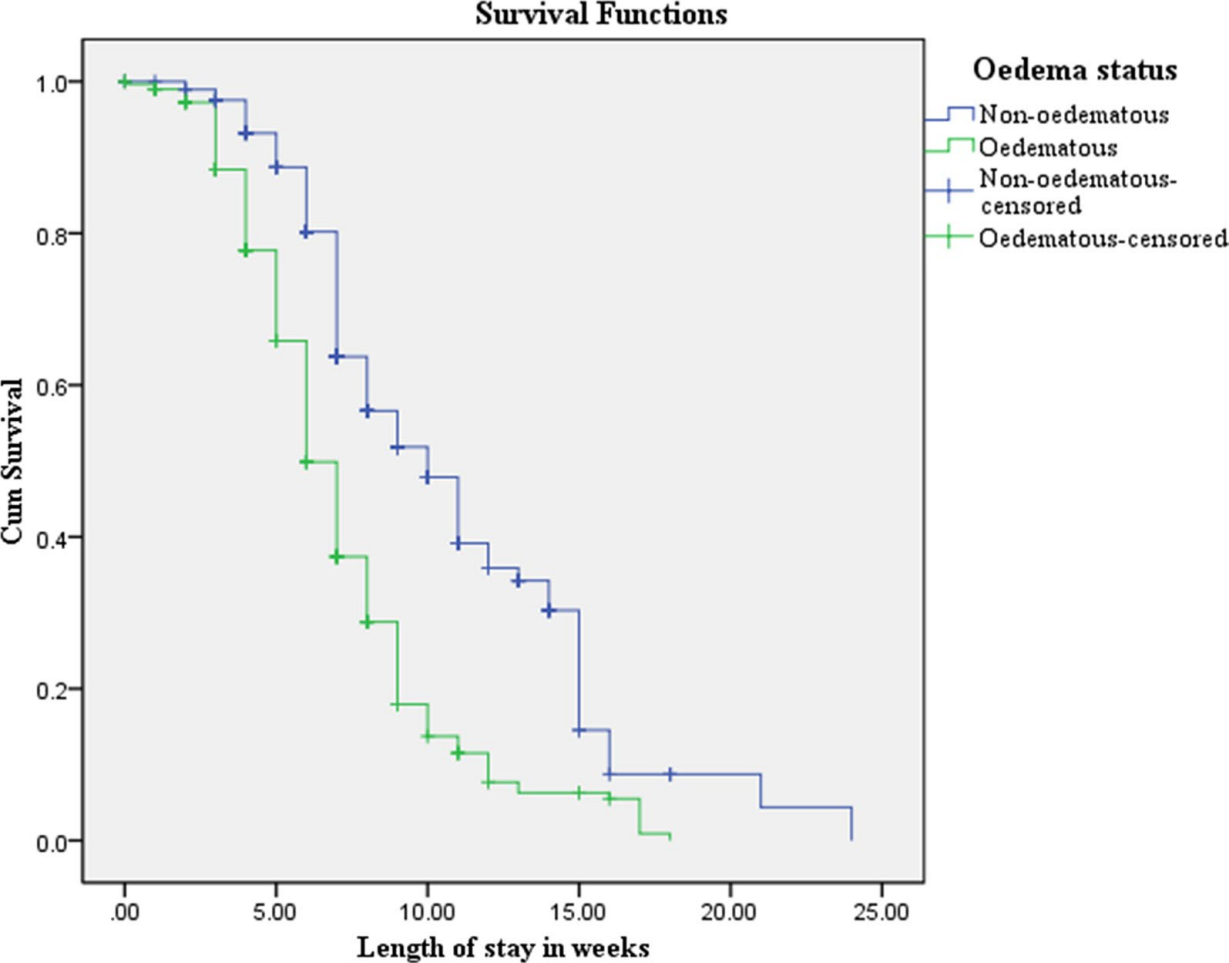
The percentage of survival and length of stay in weeks for children treated at OTP

On overage (SD) oedematous children stayed on admission for 6.4 (3.2) weeks and it was 8.1 (3.7) weeks for children admitted due to severe wasting with the mean difference of 1.7 weeks (95% CI 1.66, 2.04). The difference in the length of stay among the study arms was statistically significant (P = 0.001) (Fig. 1). Moreover, the likelihood of recovery was 2.3 times highly for oedematous children than none-oedematous ones treated at OTP (AHR = 2.25 at 95% CI 1.79, 2.82) (Table 2). This finding was in line with the some evidences arguing that none-oedematous children had slower response to the current SAM treatment as compared to the oedematous counters [23–25]. The likely reason for better recovery rate and shorter length of stay for children oedema might be due to relatively better care provided to them by caregivers and attendants.

**Table 2:** The level of significance and adjusted effect estimates of predictors on recovery rate

Variables	Categories	Treatment outcomes		P-value	AHR (95% CI)
		Recovered (%)	Not recovered (%)		
Child age	≤ 24 months	181 (45.7)	108 (58.1)	0.69	0.95 (0.74, 1.22)
	> 24 months	215 (54.3)	78 (41.9)		1
Child sex	Female	219 (55.3)	109 (58.6)	0.62	1.05 (0.86, 1.29)
	Male	177 (44.7)	77 (41.4)		1
Oedema status	Oedematous	235 (59.3)	59 (31.7)	0.000	2.25 (1.79, 2.82)*
	Non-oedematous	161 (40.7)	127 (68.3)		1
Breastfeeding status	Breastfeeding	124 (31.8)	68 (37.6)	0.72	1.04 (0.81, 1.34)
	Not breastfeeding	266 (68.2)	113 (62.4)		1
Deworming provision	Provided	78 (19.7)	26 (14.0)	0.25	0.74 (0.44, 1.24)
	Not provided	318 (80.3)	160 (86.0)		1

* Highly significant association

Oedema have been recognized as major concern to caregivers since early years; thus caregivers have more concern to oedematous malnutrition than that of severe wasting [10]. In contrast, the OTP is currently facing challenges including the commonly practiced sharing of therapeutic foods with family members [8, 20]. The reasons for sharing were related to perceptions of caregiver’s on SAM and household food insecurity [26]. Thus the curious caregivers to the oedematous SAM as they perceive it would difficult to treat [10] might have less likelihood of sharing therapeutic foods provided to their children. Therefore, we would argue that the possible reason for the better recovery of oedematous SAM could be better utilization of provided therapeutic foods.

## Conclusions

The type of SAM on admission affects the treatment outcomes of the children in outpatient therapeutic program in Boloso sore district. Oedematous children recovered better in time than those severely wasted. This could be related to better utilization of therapeutic foods by oedematous children due to caregivers’ perception towards oedema on their children. We recommend continual awareness creation on the effects of both types of SAM as they are cause or underlying factors of child deaths in Ethiopia. Targeted community sensitization on the severity of none-oedematous SAM might enhance proper use of therapeutic foods. Furthermore, monitoring proper usage of RUTF should be strengthened and should also target caregivers of none-oedematous children.

### Limitations

Lack of evidence on utilization of provided RUTF on patient card and presence of missing information in OTP cards.

## Additional file

**Additional file 1.** Sociodemographic and related characteristics of children admitted to OTP.

## Abbreviations

CIH: Centre for International Health
CMAM: Community based Management of Acute Malnutrition
NORHED: Norwegian Programme for Capacity Development in Higher Education and Research for Development
OTP: Outpatient Therapeutic Program
RUTF: ready to use therapeutic food
SAM: severe acute malnutrition
SC: Stabilization Centre
SENUPH: South Ethiopia Network of Universities in Public Health
UoB: University of Bergen
WSU: Wolaita Sodo University
