# Supplementary material for: Children with oedema recover better than those with severe wasting in outpatient therapeutic program at Boloso Sore district, Southwest Ethiopia
Source: BMC Res Notes. 2018 Feb 9;11:118. doi: 10.1186/s13104-018-3232-x (PMC5807849; doi:10.1186/s13104-018-3232-x)
Supplement: Supplementary file 1 — Additional file 1. Sociodemographic and related characteristics of children admitted to OTP. [file 13104_2018_3232_MOESM1_ESM.docx]

| Variables | Categories | Descriptions Type of SAM | |
| --- | --- | --- | --- |
|  |  | Oedematous (%) | Non-oedematous (%) |
| Child age | < 24 months | **82(27.9)** | 207(71.9) |
|  | > 24 months | **212(72.1)** | 81(28.1) |
| **Child sex** | Female | **163(55.4)** | 165(57.3) |
|  | Male | **131(44.6)** | 123(42.7) |
| **Appetite test** | Passed | **284(96.6)** | 283(98.3) |
|  | Failed | **10(3.4)** | 5(1.7) |
| **Breastfeeding** | Yes | **55(18.7)** | 137(47.6) |
|  | No | **234(79.6)** | 145(50.3) |
| Deworming provision | Provided | **67(22.8)** | 37(12.8) |
|  | Not provided | **227(77.2)** | 251(87.2) |

**Additional file 1:** Sociodemographic and related characteractics of children admitted to OTP
